# Yupingfeng polysaccharides enhances growth performance in Qingyuan partridge chicken by up‐regulating the mRNA expression of SGLT1, GLUT2 and GLUT5

**DOI:** 10.1002/vms3.167

**Published:** 2019-04-11

**Authors:** Fuquan Yin, Ruixia Lan, Zhengmin Wu, Zhijing Wang, Haohao Wu, Zhiming Li, Hui Yu, Zhihui Zhao, Hua Li

**Affiliations:** ^1^ Department of Animal Science College of Agriculture Guangdong Ocean University Zhanjiang Guangdong P.R. China; ^2^ School of Life Science and Engineering Foshan University Foshan Guangdong P.R. China

**Keywords:** Yupingfeng polysaccharides, growth performance, intestine morphology, SGLT1, GLUT2 and GLUT5, Qingyuan partridge chicken

## Abstract

The ban on the use of antibiotic in feed encouraged nutritionists to using alternatives to maintain growth performance and intestinal function of broilers. This study was conducted to evaluate the effects of Yupingfeng polysaccharides (YP) supplementation on growth performance and expression of SGLT1, GLUT2 and GLUT5 in Qingyuan partridge chicken. Experiment 1: a total of 540 chickens were randomly allocated to five groups with six replication. Dietary treatments were: (1) CON (control group), basal diet; (2) T1, CON + 0.5 g kg^−1^ YP; (3) T2, CON + 1 g kg^−1^ YP; (4) T3, CON + 2 g kg^−1^ YP; (5) T4, CON + 4 g kg^−1^ YP. Experiment 2, a total of 162 were randomly allocated to three groups with three replication. Dietary treatments were: (1) CON, basal diet; (2) T1, CON + 0.5 g kg^−1^ YP; (3) T2, CON + 1 g kg^−1^ YP. From days 1 to 14 and overall, chicken fed T1 diet had higher ADG. On day 42, there was increased villus height of jejunum in T1 group. On days 14 and 28, there was decreased villus height of duodenum and jejunum in T2 group. In duodenum, the expression of SGLT1 (days 21, 35 and 42), GLUT2 (days 7, 14, 21, 28, 35 and 42) and GLUT5 (days 7, 14, 21 and 28) was increased with YP supplementation. In jejunum, the expression of SGLT1 (days 7, 14, 21, 28 and 35), GLUT2 (days 14, 21, 28, 35 and 42) and GLUT5 (days 7, 14, 21, 28, 35 and 42) was increased with YP supplementation. In ileum, the expression of SGLT1 (days 7, 21, 35 and 42), GLUT2 (days 7, 14, 21 and 42) and GLUT5 (days 7, 14, 21, 28, 35 and 42) was increased with YP supplementation. Dietary YP supplementation improves growth performance and expression of SGLT1, GLUT2 and GLUT5 in intestine.

## Introduction

The prophylactic use of antibiotics in poultry nutrition to improve growth performance and decrease mortality from clinical diseases is well documented (Dibner & Richards 2005). However, the growing concern over the transmission and the proliferation of resistant bacteria via the food chain have led to the ban of antibiotic as growth promoters in livestock production in European Union since 2006 (Castanon 2007; Asai *et al*. 2011; Dheilly *et al*. 2011). Antibiotic alternatives to enhance animal production and health status are a topic of concerted interest. Particular interest is now being paid to the phytogenic feed additives due to their plant‐derived properties, growth promoting effects, antimicrobial and antioxidant activities as well as digestion and immune enhancing properties (Windisch *et al*. 2008; Brenes & Roura 2010; Puvaca *et al*. 2013; Cerisuelo *et al*. 2014; Gong *et al*. 2014).

The gastrointestinal tract is the main site of nutrient absorption and the main place of chemosensory system (Furness *et al*. 2013). The gut chemosensory system can regulate digestion, absorption and metabolism, and has nutritional and pharmacological applications in improving gut development and health (Mace & Marshall 2013). Gut epithelium has approximately 90% of the absorptive epithelial cells with the expression of nutrient transporters (Henning *et al*. 1994). The main function of nutrient transporters is to absorb nutrients from the lumen of gut. It has been reported that phytogenic compounds can regulate the gene expression profile of ileal mucosa (Liu *et al*. 2013a, 2014). The monosaccharides absorption in the small intestine mainly depends on three transporters (Roohollah *et al*. 2015), Na^+^‐dependent glucose and galactose transporter (SGLT1), Na^+^‐independent glucose, galactose and fructose transporter (GLUT2) and Na^+^‐independent fructose transporter (GLUT5). Glucose absorption in the intestine has an important role in maintain cellular and organic functions (Jane *et al*. 2003), and the expression levels of intestine glucose transporters are crucial to the absorption and uptake of glucose in the small intestine (Rodriguez *et al*. 2004), but there has no study of the effects of Yupingfeng polysaccharides (YP) on expression of SGLT1, GLUT2 and GLUT5.

YP is a traditional Chinese medicine decoction, which is composed of three herbs, Astragali Radix (AR, Huangqi), Atractylodis Macrocephalae Rhizoma (AMR, Baizhu) and Saposhnikoviae Radix (SR, FangFeng). According to Chinese medicine principles, this decoction is used to treat colds, flus and inflammation‐associated diseases. Recent studies demonstrated that YP exerts antiviral effects including effects against influenza virus, human respiratory syncytial virus and severe acute respiratory syndrome (SARS) virus (Chiu *et al*. 2009; Liu *et al*. 2013b), as well as curative effects in inflammation‐associated diseases including allergic rhinitis (Makino *et al*. 2004) and asthma (Fang *et al*. 2005). However, no study was done to evaluate the effects of the YP on small intestine morphology and nutrient absorption in the intestine of Qingyuan partridge chicken. Therefore, the aim of this study was to investigate the effects of YP on growth performance, small intestine morphology and monosaccharides absorption transporters (SGLT1, GLUT2 and GLUT5) in Qingyuan partridge chicken, and explore the regulative mechanism for YP on growth performance of Qingyuan partridge chicken.

## Materials and methods

The experimental protocol used in this study was approved by the Animal Care and Use Committee of Guangdong Ocean University.

### Yupingfeng polysaccharide product

The commercial YP product used in this study was provided by Dezong Pharmaceutical Co., Ltd, Foshan, China. In the preparation of YP, the amounts of crude drugs (in slices) of AR, AMR and SR were weighed according to the weight ratio of 3:1:1, separately. The herbal mixture was boiled in 8 volumes of water (v/w) by moderate heating for 2 h. The residues were re‐boiled in 6 volumes of water for 1 h. The extracts pooled from two extractions were filtered, and which were dried by lyophilization and stored at 4°C. The carbohydrate and protein concentrations of YP were 34.20% and 18.52%, respectively.

### Experimental design, animals and housing

Experiment 1, a total of 540 1‐day‐old Qingyuan partridge chicken (Guangdong Tiannong Food Ltd, Guangdong, China) were used in this 28‐day experiment. All chickens were individually weighed and randomly allocated to five groups (108 chickens/group) with six replication (18 chickens/pen) according to initial body weight. Dietary treatments were: (1) CON (control group), basal diet; (2) T1, CON + 0.5 g kg^−1^ YP; (3) T2, CON + 1 g kg^−1^ YP; (4) T3, CON + 2 g kg^−1^ YP; (5) T4, CON + 4 g kg^−1^ YP.

Experiment 2, a total of 162 1‐day‐old Qingyuan partridge chicken were purchased from the same company to a 42‐day experiment. All chickens were individually weighed and randomly allocated to three groups (54 chickens/group) with three replication (18 chickens/pen) according to initial body weight. Dietary treatments were: (1) CON, basal diet; (2) T1, CON + 0.5 g kg^−1^ YP; (3) T2, CON + 1 g kg^−1^ YP. The diet was formulated to meet or exceed the nutritional requirements of chickens during starter (days 1 to 21) and grower (days 22 to 42) phases, according to the NRC (National research council, 1998) recommendations (Table 1). All chickens were placed in battery cages (80 cm length × 80 cm width × 35 cm height). The temperature of the room was maintained at 33 ± 1°C for the first week. From day 8, the temperature was gradually reduced by 0.5°C per day until maintained at 24°C. Artificial light was provided 24 h d^−1^ by fluorescent lights.

**Table 1:** Composition of basal diets (as‐fed basis)

Item, %	d 1 to 21	d 22 to 45
Ingredient		
Corn	67.6	70.4
Soybean meal	28.0	25.0
Soybean oil	0.5	0.8
Dicalcium phosphate	1.3	1.3
Limestone	1.1	1.1
L‐Lysine	0.3	0.3
DL‐Methionine	0.2	0.2
Premix^1^	1.0	1.0
Total	100	100
Calculated composition		
Metabolizable energy (MJ kg^−1^)	12.14	12.35
Crude protein	20.0	19.0
Lysine	1.2	1.0
Methionine	0.6	0.5
Methionine + Cystine	0.9	0.8
Calcium	1.0	0.86
Available phosphorus	0.4	0.4

^1^Provided per kg of complete diet: vitamin A, 8000 IU; vitamin D_3_, 2000 IU; vitamin E, 50 mg; vitamin B_1_, 2 mg; vitamin B_2_, 5 mg; vitamin B_12_, 0.3 mg; niacin, 40 mg; d‐pantothenic acid, 15 mg; folic acid, 1 mg; choline, 800 mg; Fe (as FeSO_4_·7H_2_O), 80 mg; Cu (as CuSO_4_·5H_2_O), 12 mg; Zn (as ZnSO_4_), 85 mg; Mn (as MnO_2_), 8 mg; I (as KI), 0.28 mg; and Se (as Na_2_SeO_3_·5H_2_O), 0.15 mg.

### Experimental procedures and sampling

In experiment 1, chickens were weighed on a pen basis on days 1, 15 and 28, and feed consumption was recorded on a pen basis throughout the experiment to calculate the average daily gain (ADG), average daily feed intake (ADFI) and feed conversion ratio (FCR).

In experiment 2, after 12 h fasting and free access to feed, on days 14, 28 and 42, two chickens were randomly selected from each pen, the chickens were euthanized with electricity and their abdominal cavities were opened to remove the gastrointestinal tract. The small intestine was carefully dissected from the mesentery, and carefully divided into three parts, duodenum (from the end of the gizzard to pancreatic loop), jejunum (from the pancreatic loop to Meckel's diverticulum) and ileum (from Meckel's diverticulum to the ileocecal junction), respectively. After measuring the length of duodenum, jejunum and ileum, 1 cm segments were obtained from duodenum, jejunum and ileum (the middle of each part). These segments were placed in 4% neutral formalin for histological analysis. On days 7, 14, 21, 28, 35 and 42, after feeding 2 h, one chicken was randomly selected from each pen, the chickens were euthanized with electricity and their abdominal cavities were opened to remove the gastrointestinal tract, 2 cm segments were obtained from duodenum, jejunum and ileum (the middle of each part), then rinsed with RNase‐free PBS and frozen at 80°C for total RNA extraction.

### Histology

The samples were fixed in 4% neutral formalin for 72 h at room temperature and subsequently dehydrated through a graded ethanol series and then cleared with xylene and finally embedded in paraffin. Serial tissue sections were cut at 4 *μ*m thickness and six non‐successive sections mounted on each slide (Fisher Scientific, USA). The sections were deparaffinized, rehydrated and rinsed in distilled water. Finally, they were stained with haematoxylin for 5 min and then eosin for 40 s, dehydrated and mounted.

The sections were examined under a light microscope (Olympus BX‐51, Japan) with image analysis software (Image‐Pro Plus 6.0). Five well‐oriented villi height (Vh, determined as the distance between the crypt openings and the end of the villi) and their associated crypt depth (Cd, measured from the crypt‐villous junction to the base of the crypt) per section was measured under a microscope at 40× magnification. The means of these measurements were calculated to yield a single value per chicken. These procedures were conducted by an observer unaware of the treatment assignment.

### Real‐time PCR

Total RNA was extracted from duodenum, jejunum and ileum with Trizol according to the manufacturer's instructions (TaKaRa, Dalian, China). The collection supernatant (400 *μ*L) was precipitated with 400 *μ*L isopropanol, followed by washing with pre‐cooled 75% ethanol. The precipitated total RNA samples were dried and then dissolve in 1 mL RNase‐free dH_2_O. The amount and purity of the total RNA were quantified by measuring the optical density at 260 and 280 nm; the RNA integrity was checked by 1.5% agarose gel electrophoresis. The cDNA synthesis was performed using a PrimerScript RT reagent kit with gDNA Eraser (TaKaRa) according to the manufacturer's instructions. First, the genomic DNA elimination reaction was performed. The 10 mL reaction solution contained 2 *μ*L 5× gDNA Eraser Buffer, 1 *μ*L gDNA Eraser, 1 *μ*g total RNA and 4 *μ*L RNase‐freed H_2_O. The reaction proceeded at room temperature for 5 min. Then, 4 *μ*L 5 × PrimerScript buffer, 1 *μ*L 5 × PrimerScript RT enzyme mix, 1 *μ*L RT primer mix and 4 *μ*L RNase‐free dH_2_O were added to the solution. The reaction was performed in a programmable thermal controller PTC‐100 (MJ Research Inc.) and incubated at 37°C for 15 min, followed by heat inactivation at 85°C for 5 s. Real‐time PCR analysis for SGLT1, GLUT2 and GLUT5 expression in duodenum, jejunum and ileum was performed using a CFX‐96 (Bio‐Rad). The 20 *μ*L real‐time PCR solution mixture contained 10 *μ*L SYBR premix, 0.8 *μ*L sense and antisense primers (stock concentration 10 mmol L^−1^), 6.8 *μ*L water and 1.6 *μ*L cDNA. The primers used are listed in Table 2. The *β*‐actin, SGLT1, GLUT2 and GLUT5 mRNA sequences were obtained from the Genebank of the National Center for Biotechnology Information of the National Institutes of Health (http://www.ncbi.nlm.nih.gov/cgi-bin/genbank). The specific primers were designed using Primer Premier 5.0 software. The annealing temperature for each primer set is given in Table 2. The real‐time PCR was performed with an initial incubation at 95°C for 30 s, followed by 40 cycles at 95°C for 5 s, and then annealing for 30 s; during this period, the real‐time fluorescence data were collected. A melting‐curve protocol was performed by repeating the 95°C heating for 10 s, from 65°C to 95°C with 0.5°C increments, during which the fluorescence data were collected to evaluate the specific amplification. The relative differences for a gene in the different groups were determined using the comparative cycle threshold (∆∆C_T_) method (Livak & Schmittgen 2001). The resulting values were converted to fold changes compared with the *β*‐actin signal by raising two to the −∆∆C_T_ power (2^−∆∆CT^). To confirm the product's specificity, each PCR product was analysed by 1.5% agarose gel electrophoresis followed by sequencing to confirm that these were the correct DNA fragments.

**Table 2:** Primers used for real‐time PCR

Gene names	Product length (bp)	Sequences (5′‐3′)	Annealing temperature (°C)	Accession number
*β*‐actin	177	F: GAGCTATGAACTCCCTGATG	57	NM_205518.1
		R: GTGTTGGCATACAGATCCTT		
SGLT1	232	F: 5′‐TCAGGTCTACCTGTCAATCC	57	NM_001293240.1
		R: GAGAATGAAAGATCCCACAA		
GLUT2	113	F: CCGCAGAAGGTGATAGAAGC	57	NM_207178.1
		R: CCTGGGATGGTGACAGTGAT		
GLUT5	166	F: ATTGTTGCTGCAGTCCTTAT	57	XM_004947448.1
		R: CTATCCCAATAGCACCTCTG		

### Statistical analysis

The data were analysed by one‐way ANOVA. Duncan's multiple comparison was used for the detection of significant difference using SAS 9.1 software. *P*‐value less than 0.05 was considered to be a significant difference.

## Results

### Growth performance

The effects of YP supplementation on growth performance were presented in Table 3. From days 1 to 14, chickens fed T1 and T4 diets had higher (*P* *<* 0.05) ADG than chickens fed CON diet, no significant differences were observed in ADFI or FCR. From days 15 to 28, no significant differences were observed in ADG, ADFI or FCR. The overall period, chickens fed T1 diet had higher (*P* *<* 0.05) ADG than chickens fed CON diet, no significant differences were observed in ADFI or FCR.

**Table 3:** Effects of YP supplementation on growth performance in Qingyuan partridge chicken^1^

Items	CON	T1	T2	T3	T4
d 1 to 14					
ADG, g	5.50 ± 0.01^a^	5.69 ± 0.11^b^	5.64 ± 0.03^ab^	5.64 ± 0.05^ab^	5.70 ± 0.05^b^
ADFI, g	9.824 ± 0.06	9.92 ± 0.03	10.17 ± 0.15	10.10 ± 0.19	10.09 ± 0.06
FCR	1.79 ± 0.01	1.75 ± 0.04	1.81 ± 0.03	1.79 ± 0.02	1.77 ± 0.03
d 15 to 28					
ADG, g	8.75 ± 0.08	9.27 ± 0.28	9.17 ± 0.25	9.11 ± 0.13	9.17 ± 0.12
ADFI, g	18.72 ± 0.13	19.11 ± 0.31	19.04 ± 0.12	18.96 ± 0.14	18.71 ± 0.10
FCR	2.17 ± 0.02	2.07 ± 0.09	2.07 ± 0.05	2.15 ± 0.41	2.11 ± 0.04
Overall					
ADG, g	7.03 ± 0.08^a^	7.44 ± 0.06^b^	7.30 ± 0.11^ab^	7.19 ± 0.18^ab^	7.17 ± 0.15^ab^
ADFI, g	14.06 ± 0.09	14.31 ± 0.18	14.39 ± 0.03	14.38 ± 0.06	14.10 ± 0.17
FCR	1.20 ± 0.02	1.92 ± 0.04	1.97 ± 0.03	2.00 ± 0.05	1.98 ± 0.02

^1^Abbreviation: CON, Basal diet; T1, CON + 0.5 g kg^−1^ YP; T2, CON + 1 g kg^−1^ YP; T3, CON + 2 g kg^−1^ YP; T4, CON + 4 g kg^−1^ YP.

^a,b^Different letter superscripts mean significant difference (*P* *<* 0.05).

### Intestine length

The effects of YP supplementation on intestine length were presented in Table 4. On days 14, 28 and 42, no significant difference was observed in duodenum length, jejunum length or ileum length.

**Table 4:** Effects of YP supplementation on intestine length in Qingyuan partridge chicken^1^

Items	CON	T1	T2
d 14			
Duodenum, cm	16.09 ± 0.63	16.32 ± 1.02	16.76 ± 1.34
Jejunum, cm	16.35 ± 1.66	17.78 ± 1.83	16.83 ± 1.06
Ileum, cm	22.92 ± 1.93	22.74 ± 0.60	23.33 ± 1.17
d 28			
Duodenum, cm	16.20 ± 0.53	16.95 ± 0.55	17.05 ± 0.64
Jejunum, cm	27.57 ± 1.63	28.65 ± 0.91	28.33 ± 1.47
Ileum, cm	28.42 ± 1.09	28.29 ± 1.05	27.20 ± 0.58
d 42			
Duodenum, cm	20.35 ± 0.85	20.63 ± 0.65	21.25 ± 1.34
Jejunum, cm	29.63 ± 0.62	29.65 ± 0.49	29.97 ± 0.52
Ileum, cm	28.44 ± 1.15	27.10 ± 0.55	28.18 ± 1.41

^1^ Abbreviation: CON, Basal diet; T1, CON + 0.5 g kg^−1^ YP; T2, CON + 1 g kg^−1^ YP.

### Intestine morphology

The effects of YP supplementation on intestine structure were presented in Table 5. On day 14, villus height in duodenum was higher (*P* *<* 0.05) in CON and T1 groups compared with T2 group. Villus height in jejunum was higher (*P* *<* 0.05) in CON group compared with T2 group. On day 28, in duodenum, villus height was higher (*P* *<* 0.05) in CON group compared with T2 group. In jejunum, crypt depth was lower (*P* *<* 0.05) in CON group compared with T1 group. On day 42, in jejunum, villus height was lower (*P* *<* 0.05) in CON group compared with T1 group, crypt depth was lower (*P* *<* 0.05) in T1 group compared with T2 group.

**Table 5:** Effects of YP supplementation on intestinal morphology in Qingyuan partridge chicken^1^

Items	CON	T1	T2
d 14			
Duodenum, *μ*m			
Villus height	926.11 ± 48.91^a^	905.57 ± 18.77^a^	716.94 ± 16.29^b^
Crypt depth	241.83 ± 15.43	232.65 ± 14.18	213.04 ± 5.32
Jejunum, *μ*m			
Villus height	574.89 ± 16.32^a^	511.27 ± 25.99^ab^	505.07 ± 18.02^b^
Crypt depth	88.38 ± 2.23	92.91 ± 12.97	86.83 ± 8.30
Ileum, *μ*m			
Villus height	415.87 ± 59.19	564.52 ± 56.92	569.23 ± 67.39
Crypt depth	185.41 ± 17.74	225.70 ± 7.32	221.46 ± 17.80
d 28			
Duodenum, *μ*m			
Villus height	1098.17 ± 23.88^a^	1045.74 ± 25.37^ab^	1019.49 ± 16.38^b^
Crypt depth	217.98 ± 11.21	205.32 ± 8.36	201.13 ± 32.86
Jejunum, *μ*m			
Villus height	720.63 ± 17.02	636.43 ± 64.38	722.01 ± 46.84
Crypt depth	83.60 ± 8.13^a^	118.58 ± 13.84^b^	104.72 ± 8.34^ab^
Ileum, *μ*m			
Villus height	547.79 ± 42.03	550.08 ± 79.12	631.06 ± 63.04
Crypt depth	122.80 ± 26.13	111.44 ± 6.00	136.20 ± 18.99
d 42			
Duodenum, *μ*m			
Villus height	1101.54 ± 48.56	996.85 ± 40.46	959.00 ± 47.23
Crypt depth	89.28 ± 6.50	86.40 ± 1.62	88.70 ± 11.91
Jejunum, *μ*m			
Villus height	823.40 ± 15.28^a^	903.74 ± 24.63^b^	863.22 ± 12.25^ab^
Crypt depth	88.48 ± 6.16^ab^	79.32 ± 5.76^a^	93.39 ± 3.16^b^
Ileum, *μ*m			
Villus height	586.12 ± 20.24	635.43 ± 16.01	598.65 ± 17.43
Crypt depth	78.09 ± 9.20	69.60 ± 4.74	71.41 ± 5.58

^1^Abbreviation: CON, Basal diet; T1, CON + 0.5 g kg^−1^ YP; T2, CON + 1 g kg^−1^ YP.

^a,b^Different letter superscripts mean significant difference (*P* *<* 0.05).

### SGLT1, GLUT2 and GLUT5 mRNA expression

**Figure 1:**
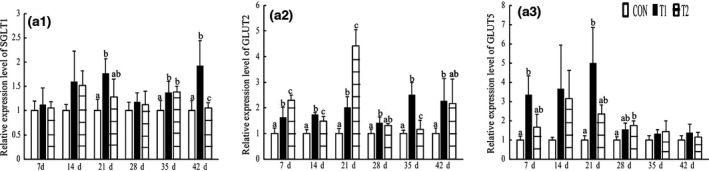
Changes in the expression level of SGLT1, GLUT2 and GLUT5 in duodenum with YP supplementation. SGLT1, Na^+^‐dependent glucose and galactose transporter; GLUT2, Na^+^‐independent glucose, galactose and fructose transporter; GLUT5: Na^+^‐independent fructose transporter. ^a,b,c^Different letter superscripts mean significant difference (*P* *< *0.05).

**Figure 2:**
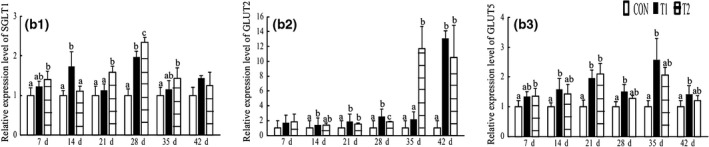
Changes in the expression level of SGLT1, GLUT2 and GLUT5 in jejunum with YP supplementation. SGLT1, Na^+^‐dependent glucose and galactose transporter; GLUT2, Na^+^‐independent glucose, galactose and fructose transporter; GLUT5, Na^+^‐independent fructose transporter. ^a,b,c^Different letter superscripts mean significant difference (*P* *<* 0.05).

**Figure 3:**
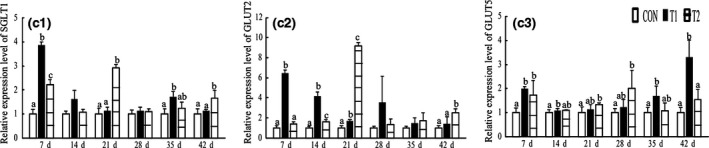
Changes in the expression level of SGLT1, GLUT2 and GLUT5 in ileum with YP supplementation. SGLT1: Na^+^‐dependent glucose and galactose transporter; GLUT2: Na^+^‐independent glucose, galactose and fructose transporter; GLUT5: Na^+^‐independent fructose transporter. ^a,b,c^Different letter superscripts mean significant difference (*P* *<* 0.05).

The effects of YP supplementation on SGLT1, GLUT2 and GLUT5 expression in duodenum, jejunum and ileum were presented in Figs 1, 2, 3, respectively.

In duodenum, on day 21, the expression of SGLT1 mRNA was lower (*P* *<* 0.05) in CON group compared with T1 group. On day 35, the expression of SGLT1 mRNA was lower (*P* *<* 0.05) in CON group compared with T1 and T2 groups. On day 42, the expression of SGLT1 mRNA was lower (*P* *<* 0.05) in CON and T2 groups compared with T1 group. The expression of GLUT2 mRNA was lower (*P* *<* 0.05) in CON group compared with T1 and T2 groups on days 7, 14, 21 and 35. On days 28 and 42, the expression of GLUT2 mRNA was lower (*P* *<* 0.05) in CON group compared with T1 group. The expression of GLUT5 mRNA was lower (*P* *<* 0.05) in CON group compared with T1 group on days 7 and 21. On day 28, the expression of GLUT5 mRNA was lower (*P* *<* 0.05) in CON group compared with T2 group.

In jejunum, on days 7 and 35, the expression of SGLT1 mRNA was lower (*P* *<* 0.05) in CON group compared with T2 group. On day 14, the expression of SGLT1 mRNA was lower (*P* *<* 0.05) in CON and T2 groups compared with T1 group. On day 21, the expression of SGLT1 mRNA was lower (*P* *<* 0.05) in CON and T1 groups compared with T2 group. On day 28, the expression of SGLT1 mRNA was lower (*P* *<* 0.05) in CON group compared with T1 and T2 groups. The expression of GLUT2 mRNA was lower (*P* *<* 0.05) in CON group compared with T1 group on day 14. On days 21, 28 and 42, the expression of GLUT2 mRNA was lower (*P* *<* 0.05) in CON group compared with T1 and T2 groups. On day 35, the expression of GLUT2 mRNA was lower (*P* *<* 0.05) in CON and T1 groups compared with T2 group. The expression of GLUT5 mRNA was lower (*P* *<* 0.05) in CON group compared with T2 group on day 7. On days 14, 28, 35 and 42, the expression of GLUT5 mRNA was lower (*P* *<* 0.05) in CON group compared with T1 group. On day 21, the expression of GLUT5 mRNA was lower (*P* *<* 0.05) in CON group compared with T1 and T2 groups.

In ileum, on day 7, the expression of SGLT1 mRNA was lower (*P* *<* 0.05) in CON group compared with T1 and T2 group. On days 21 and 42, the expression of SGLT1 mRNA was lower (*P* *<* 0.05) in CON and T1 groups compared with T2 group. On day 35, the expression of SGLT1 mRNA was lower (*P* *<* 0.05) in CON group compared with T1 group. The expression of GLUT2 mRNA was lower (*P* *<* 0.05) in CON and T2 groups compared with T1 group on day 7. On days 14 and 21, the expression of GLUT2 mRNA was lower (*P* *<* 0.05) in CON group compared with T1 and T2 groups. On day 42, the expression of GLUT2 mRNA was lower (*P* *<* 0.05) in CON and T1 groups compared with T2 group. The expression of GLUT5 mRNA was lower (*P* *<* 0.05) in CON group compared with T1 and T2 groups on day 7. On days 14 and 35, the expression of GLUT5 mRNA was lower (*P* *<* 0.05) in CON group compared with T1 group. On days 21 and 28, the expression of GLUT5 mRNA was lower (*P* *<* 0.05) in CON group compared with T2 group. On day 42, the expression of GLUT5 mRNA was lower (*P* *<* 0.05) in CON and T2 groups compared with T1 group.

## Discussion

There are a limited number of controlled studies on the effects of phytogenic compounds on growth performance in broiler chickens. In this study, YP has beneficial effects on ADG during days 1 to 14 and days 1 to 28, but has no effect on ADFI or FCR. This is in agreement with Ocak *et al*. (2008), who reported the peppermint leaves had a higher body weight gain in broilers during days 7 to 21 and days 7 to 35. Hernández *et al*. (2004) also reported that the plant extract had higher weight gain during days 14 to 21. The results are not always consistent, Amad *et al*. (2011) reported that phytogenic compounds improved FCR, but had no effects on body weight gain. Chen *et al*. (2003) reported that astragalin and achyranthan had no effects on ADG, ADFI or FCR. The results from previous studies are highly variable. There appear to be three reasons associated with the inconsistency: (1) variations in the composition of phytogenic compounds due to plant growing locations and extraction methods (Yang *et al*. 2009); (2) variations in the dosages that may not be efficacious (Cross *et al*. 2007); (3) varied conditions during the trials such as environment, animal age, genetics, feeds and health status (Giannenas *et al*. 2003).

The effects of YP were not significant on the duodenum, jejunum and ileum length on days 14, 28 and 42. This is in agreement with Ocak *et al*. (2008), who reported the supplementation of peppermint and thyme had no significant effects on gut length and gut weight.

Morphological changes in small intestinal caused by YP may provide further information on possible benefits to the digestive tract. A significant increase in villous height in the jejunum was observed on day 42 with 0.5 g kg^−1^ YP supplementation compared to the control group, indicating a certain rapid maturation of the intestines. Our result was supported by Murugesan *et al*. (2015), who reported the phytogenic feed additives increased the villus height of jejunum and ileum on day 39. The increase in villus height demonstrated improved architecture and active sites between the liminal digesta and the epithelium, which might result in an increased digestive and absorptive ability (Murugesan *et al*. 2013). The beneficial effect of YP in increasing the villus height in the jejunum could increase the absorptive efficiency, because jejunum is the main organ for nutrient absorption. Additionally, the greater villus height increases the activity of mucosal digestive enzymes, resulting in improved digestibility (Baurhoo *et al*. 2007; Murugesan *et al*. 2014). Intestinal crypts are the source of epithelial cells for villi, and crypt depth is directly correlated with epithelial cell turnover. In this study, the smaller crypt height of jejunum on day 42 with 0.5 g kg^−1^ YP supplementation compared to the control group, although the reduction did not reach statistical significance, the shallower crypts in the jejunum suggesting the decreased cellular turnover and improved health intestine. In addition, cellular turnover is an energy consuming process that uses resources that may otherwise be utilized towards growth, thus, shallower crypts are also related to improve growth performance (Markovic *et al*. 2009). However, on day 28, the crypt height of jejunum with 0.5 g kg^−1^ YP was higher compared to the CON group. On days 14 and 28, the villus height of duodenum was decreased with 1 g kg^−1^ YP compared to the CON group. The available literatures also cannot provide a consistent result. Former literatures have shown increased, unchanged and reduced villi length and crypt depth in the jejunum and colon for broilers and pigs with phytogenic feed additives (Namkung *et al*. 2004; Demir *et al*. 2005; Jamroz *et al*. 2006; Nofrarias *et al*. 2006; Oetting *et al*. 2006). So it is difficult to conclude that the relevance of changes in intestinal morphology in growth promoting potential of phytogenic feed additives.

Glucose uptake is mediated by SGLT1 in the brush‐border membrane, and by passive permeation through the paracellular route. GLUT 2 transports glucose and fructose out of the enterocyte across the basolateral membrane (Jane *et al*. 2003; Breves *et al*. 2007). GLUT5 is the only member specific to fructose (Kayano *et al*. 1990; Burant *et al*. 1992) and together with GLUT2, which transports fructose in addition to glucose. The expression of SGLT1, GLUT2 and GLUT5 was crucial to the absorption and uptake of glucose in the small intestine. Song *et al*. (2009, 2010) reported the elevation of the mRNA expression of SGLT1 and GLUT2 resulted in the up‐regulation of glucose absorption. In this study, YP increased the mRNA expression of SGLT1, GLUT2 and GLUT5 in the duodenum, jejunum and ileum, and the elevation of SGLT1, GLUT2 and GLUT5 mRNA expression may help small intestinal digestion and absorption.

## Conclusion

In conclusion, 0.5 g kg^−1^ YP supplementation improved ADG during days 1 to 14 and days 1 to 28, increased the villus height of jejunum on day 42. In addition, the supplementation of YP up‐regulated mRNA expression of SGLT1, GLUT2 and mRNA expression of GLUT5 in duodenum, jejunum and ileum, which indicated that the dietary supplementation of YP improved the growth performance by up‐regulating of SGLT1, GLUT2 and GLUT5 mRNA expression.

## Acknowledgements

Authors are grateful for the support of Guangdong Provincial Key Laboratory of Animal Molecular Design and Precise Breeding. Thanks for the Lab members' their kind help during the experiment.

## Source of funding

This study was supported by the Key Program of Applied Technology Research in Guangdong Province (2016B020233007), and the Engineering Technology Research Center of Chicken Commercialized Breeding in Guangdong (2017–1649).

## Conflict of interest

No potential conflict of interest was reported by the authors.

## Ethical statement

The authors confirm that the ethical policies of the journal, as noted on the journal's author guidelines page, have been adhered to. No ethical approval was required as this is a case report with no original research data. Informed consent was obtained from the client for the publication of this case report.

## Contributions

Conceived and designed the experiments: HL and HY. Performed the experiments: ZWu, ZWang, HW and ZL. Analyzed the data: ZL. Wrote the manuscript: FY. Revised the manuscript: RL and ZZ.
